# Neurocritical care complications and interventions influence the outcome in aneurysmal subarachnoid hemorrhage

**DOI:** 10.1186/s12883-021-02054-6

**Published:** 2021-01-19

**Authors:** Alexander Hammer, Frank Erbguth, Matthias Hohenhaus, Christian M. Hammer, Hannes Lücking, Markus Gesslein, Monika Killer-Oberpfalzer, Hans-Herbert Steiner, Hendrik Janssen

**Affiliations:** 1 Department of Neurosurgery, Paracelsus Medical University, Breslauer Straße 201, 90471 Bavaria Nuremberg, Germany; 2 Department of Neurology, Paracelsus Medical University, Breslauer Str. 201, 90471 Bavaria Nuremberg, Germany; 3 Department of Anaesthesiology, Paracelsus Medical University, Breslauer Str. 201, 90471 Bavaria Nuremberg, Germany; 4 Department of Anatomy 2, University of Erlangen-Nuremberg, Universitätsstraße 19, 91054 Bavaria Erlangen, Germany; 5 Department of Neuroradiology, University of Erlangen-Nürnberg, Schwabachanlage 6, 91054 Erlangen, Germany; 6 Department of Orthopaedics and Traumatology, Paracelsus Medical University, Breslauer Str. 201, 90471 Bavaria Nuremberg, Germany; 7 Neurology/Research Institute of Neurointervention, Paracelsus Medical University, Ignaz Harrer Str. 79, Salzburg, Austria; 8 Department of Neuroradiology, Ingolstadt General Hospital, Krumenauerstraße 25, 85049 Bavaria Ingolstadt, Germany

**Keywords:** Intracranial aneurysm, Subarachnoid hemorrhage, Vascular disorders, Outcome, Neurocritical care, Decompressive craniectomy

## Abstract

**Background:**

This observational study was performed to show the impact of complications and interventions during neurocritical care on the outcome after aneurysmal subarachnoid hemorrhage (SAH).

**Methods:**

We analyzed 203 cases treated for ruptured intracranial aneurysms, which were classified regarding clinical outcome after one year according to the modified Rankin Scale (mRS). We reviewed the data with reference to the occurrence of typical complications and interventions in neurocritical care units.

**Results:**

Decompressive craniectomy (odds ratio 21.77 / 6.17 ; *p* < 0.0001 / *p* = 0.013), sepsis (odds ratio 14.67 / 6.08 ; *p* = 0.037 / 0.033) and hydrocephalus (odds ratio 3.71 / 6.46 ; *p* = 0.010 / 0.00095) were significant predictors for poor outcome and death after one year beside “World Federation of Neurosurgical Societies” (WFNS) grade (odds ratio 3.86 / 4.67 ; *p* < 0.0001 / *p* < 0.0001) and age (odds ratio 1.06 / 1.10 ; *p* = 0.0030 / *p* < 0.0001) in our multivariate analysis (binary logistic regression model).

**Conclusions:**

In summary, decompressive craniectomy, sepsis and hydrocephalus significantly influence the outcome and occurrence of death after aneurysmal SAH.

## Background

Outcome of aneurysmal subarachnoid hemorrhage (SAH) is mainly attributed to direct effects of SAH, ischemic cerebral infarction and aneurysm rebleeding [1–3]. Besides the initial clinical state, age and the aneurysm size are also known to play an important role as well as delayed cerebral ischemia [4–9].

Moreover the effect of the choice of intervention regarding the outcome has been a controversial subject for years among specialists for endovascular and microsurgical aneurysm treatment [10]. Spetzler et al. confirmed with the 6-year results of the “Barrow Ruptured Aneurysm Trial” superior outcomes of endovascular coil embolization compared to microvascular clipping regarding aneurysms of the posterior circulation, but also revealed little difference in outcome between the 2 treatment methods for anterior circulation aneurysms in the long run [11].

Comorbidities and life style risk factors also seem to influence the outcome of SAH but published results, especially regarding hypertension and smoking, remain contradictory [12, 13]. Recently published investigations found that smoking and hypertension may act as predictors regarding a good clinical outcome after aneurysmal SAH [14, 15].

The impact of treatment on intensive care units (ICU) after SAH is also recognized to be a significant factor regarding the outcome after SAH [9, 16–18]. Typical SAH related complications like hydrocephalus, pneumonia and sepsis were detected as critical determinants of outcome [9, 16, 17]. Critical care strategies are suggested which focus on maintaining normothermia, normoglycemia and prevention of anemia, as well as the implementation of infection-control measures in order to improve outcome after SAH [17, 18].

In this observational study, we investigated complications and interventions after SAH in the intensive care unit and analyzed the effect on the clinical outcome and death after one year. Further we investigated the influence of these factors on the occurrence of pneumonia and tracheostomy.

## Methods

Data were extracted and analyzed from an observational database comprising 203 cases of ruptured intracranial aneurysms from 2012 to 2017 which were treated by the departments of neuroradiology, neurology, anaesthesiology and neurosurgery at the Paracelsus Medical University Nuremberg.

Occlusion of the aneurysms was achieved by microsurgical clipping or endovascular embolization methods (sole coiling, coiling in combination with balloon or stent assisted remodelling or the use of endovascular or intrasaccular flow-diverters). Allocation of the patients to the different treatment branches was part of the standard care of the patients. Patients were included in this study, if the following criteria were fulfilled:

1. Time between aneurysm rupture and treatment < 48 hours.
2. Informed consent from the patient, a patient’s relative or the patient’s guardian.
3. Verification of SAH with cranial computed tomography (CT) or lumbar puncture and verification of an associated intracranial aneurysm diagnosed in most cases by digital subtraction angiography, alternatively by CT angiography if an immediate operation had to be performed.
4. Patient survival until completion of aneurysm treatment.

Our local institutional review board stated, that for this study no submission to the ethics committee is necessary (Ethik-Kommission der Bayerischen Landesärztekammer Mühlbaurstraße 16, 81677 München ; reference number: 2019-018 fm/h). The study was performed in accordance with the ethical standards laid down in the 1964 declaration of Helsinki and its later amendments. Informed consent of the patients or their relatives was obtained at least verbally during the initial hospital stay or during the telephone interview in the follow up because written consent was not always available in terms of the initial hospital stay or follow up. The local institutional review board approved this procedure and waived a patient informed consent, as the data is fully anonymized (Ethik-Kommission der Bayerischen Landesärztekammer, Mühlbaurstraße 16, 81677 München ; reference number: 2019-018 fm/h). One patient refused to participate in this study and therefore was excluded from our collective (1 of 204). For our analysis 203 cases of SAH were available.

Outcome was measured using the modified Rankin Scale (mRS), which was prospectively evaluated by telephone interview one year after SAH. With the usage of the mRS, occurrence of death and a follow-up time after 12 months, our methods adhere to the recommendations of Stienen et al. regarding outcome assessment in order to improve comparability of results [19]. We categorized outcome results into poor clinical outcome (mRS ≥ 3) and good clinical outcome (mRS 0–2) to evaluate the associations of intensive care complications and to make a comparison to other studies possible, which use the same dichotomization. This separation of outcome measures was commonly used in large randomized trials dealing with treatment of aneurysmal SAH [10, 11]. Moreover, we conducted another dichotomization of the outcome data (survival after one year: mRS 1–5 versus occurrence of death after one year: mRS = 6) in order to present the major impact of the occurrence of death regarding its association with some of our analyzed neurocritical care complication data. With intent of finding associations of intensive care complications or interventions with outcome parameters, we collected related data during the stay in the intensive care unit (ICU). This included the occurrence of pneumonia, sepsis, hydrocephalus, shunt placement, diabetes insipidus, tracheostomy, and decompression. Cases with hydrocephalus were initially treated with external ventricular drainages (EVD). Over the course of the stay either the EVD was removed if possible or the shunt implantation was indicated and was executed as early as possible with special awareness regarding signs of infection. There were some cases of late onset of hydrocephalus and shunt placement, which occurred after stay on intensive care. These few cases were recorded until one year after SAH via telephone interview and further medical records, if they were associated with the initial event of SAH.

Decompressive craniectomy was performed in cases of raised intracranial pressure, caused by general brain edema, intracerebral hemorrhage or cerebral ischemia, which were not sufficiently controllable by conservative intensive care treatment. Diagnostic imaging as well as clinical parameters were grounds for decompression. Decompression techniques included decompressive hemicraniectomy, bifrontal craniectomy and decompression of the posterior cranial fossa.

The “cut-off” time-point of the tracheostomy-procedure was 14 days. In terms of a conceivably longer stay on ICU (because of reasons like reduced consciousness, dysphagia or weaning-problems) the tracheostomy was executed after approximately one week (with preference of a percutaneous dilatation tracheostomy).

In part data of this patient cohort were analyzed for different aspects and published previously [15, 20–23].

### Statistics

Statistical analysis was performed using SPSS version 21 software (SPSS Inc., Cary, SC) for descriptive statistics and the results of binary logistic regression analysis. A maximum of 10 independent variables (covariates) in the binary logistic regression were included. Number of cases of every independent variable (covariate) was > 15. The Nagelkerke R Square and Hosmer and Lemeshow test value were stated along with every executed binary regression analysis. P-values below 0.05 indicated statistical significance.

## Results

Tables regarding the baseline data and the outcome data were published previously [15]. For the baseline data all 203 cases of SAH were available, outcome data were recorded from 199 to 203 patients.

Table 1 summarizes the complications (pneumonia, sepsis, hydrocephalus, diabetes insipidus) and interventions (shunt procedure because of ongoing or late onset hydrocephalus, tracheostomy, decompressive craniectomy) during stay on intensive care. A considerable part of our collective suffered from pneumonia (43.8 %) and hydrocephalus (51.7 %). Less frequent complications were sepsis (7.9 %) and diabetes insipidus (13.8 %).

**Table 1:** Complications and Interventions on Intensive Care Unit

Complications / Interventions	n	Percentage
Pneumonia	89	43.8 %
Sepsis	16	7.9 %
Hydrocephalus	105	51.7 %
Shunt	30	14.8 %
Diabetes insipidus	28	13.8 %
Tracheostomy	62	30.5 %
Decompressive craniectomy	43	21.2 %
Total	203	100.0 %

Data of complications and interventions were available for all 203 cases of SAH.

### Influence of complications and interventions on the outcome after one year

Outcome data were dichotomized in “good outcome” (mRS = 0–2) and “poor outcome” (mRS = 3–6) and used as the dependent variable in binary logistic regression analysis. Included baseline characteristics as independent variables were sex, age and WFNS grade. The Nagelkerke R Square value (0.682) and the Hosmer and Lemeshow tests (0.855) showed good quality for the model of our test regarding the prediction of poor outcome.

Age (*p* = 0.0030), WFNS grade (*p* < 0.0001), sepsis (*p* = 0.037), hydrocephalus (*p* = 0.010) and decompressive craniectomy (*p* < 0.0001) showed statistical significance regarding the prediction of outcome after one year. A very strong influence on the prediction of outcome after one year was attributed to decompressive craniectomy (odds ratio (OR) 21.77) and sepsis (OR 14.67). Strong influence was verifiable for the WFNS grade (OR 3.86) and hydrocephalus (OR 3.71). Age had a mild influence regarding the prediction of outcome after one year (OR 1.06). All other independent variables did not reach significance (Table 2).

**Table 2:** Prediction of Outcome after one Year

Risk factors	Odds ratio	95 % Confidence interval	p
Sex	1.40	0.55–3.56	0.49
Age	1.06	1.02–1.10	0.0030
WFNS Grade	3.86	2.23–6.68	< 0.0001
Pneumonia	2.68	0.925–7.75	0.069
Sepsis	14.67	1.18–181.91	0.037
Hydrocephalus	3.71	1.36–10.08	0.010
Shunt	1.04	0.25–4.36	0.96
Diabetes insipidus	0.40	0.098 -1.60	0.19
Tracheostomy	0.35	0.094–1.29	0.11
Decompressive craniectomy	21.77	4.97–95.36	< 0.0001

Binary logistic regression was performed as a multivariate analysis with dichotomized outcome (0 = “good outcome” ; 1 = “poor outcome”) as the dependent variable and sex, age, WFNS grade, pneumonia, sepsis, hydrocephalus, shunt, diabetes insipidus, tracheostomy and decompressive craniectomy as independent variables (covariates)

### Influence of complications and interventions on the occurrence of death after one year

Using binary logistic regression methods, we investigated predictors for death one year after SAH. We dichotomized status of death in our available mRS data (mRS 0–5 = “alive”; mRS 6 = “dead”). The Nagelkerke R Square value was 0.589 and the Hosmer and Lemeshow test value 0.486.

This analysis included the same significant predictors for death after one year as for the outcome after one year: Age (*p* < 0.0001), WFNS grade (*p* < 0.0001), sepsis (*p* = 0.033), hydrocephalus (*p* = 0.00095) and decompressive craniectomy (*p* < 0.013) showed statistical significance regarding the prediction of death after one year. But it also revealed additional significant predictors of survival after one year, in particular the shunting procedure (*p* = 0.025) and tracheostomy (*p* = 0.0016). Hydrocephalus (OR 6.46), sepsis (OR 6.08), decompressive craniectomy (OR 6.17) and WFNS grade (OR 4.67) had a strong influence and age a mild influence on the occurrence of death after one year (OR 1.10). With the shunting procedure (OR 0.18) and tracheostomy (OR 0.09) we revealed strong predictors of survival after one year. Sex, pneumonia and diabetes insipidus did not reach statistical significance (Table 3).

**Table 3:** Prediction of Death after one Year

Risk factors	Odds ratio	95 % Confidence interval	p
Sex	1.24	0.48–3.22	0.65
Age	1.10	1.05–1.15	< 0.0001
WFNS Grade	4.67	2.49–8.75	< 0.0001
Pneumonia	0.64	0.20–2.07	0.46
Sepsis	6.08	1.16–32.04	0.033
Hydrocephalus	6.46	2.14–19.51	0.00095
Shunt	0.18	0.042–0.81	0.025
Diabetes insipidus	0.28	0.058–1.35	0.11
Tracheostomy	0.09	0.021–0.41	0.0016
Decompressive craniectomy	6.17	1.47–25.85	0.013

Binary logistic regression was performed as a multivariate analysis with dichotomized status of death (0 = “alive”; 1 = “dead”) as the dependent variable and sex, age, WFNS grade, pneumonia, sepsis, hydrocephalus, shunt, diabetes insipidus, tracheostomy and decompressive craniectomy as independent variables (covariates)

### Influence of complications and interventions on the occurrence of pneumonia

Statistical significance regarding the prediction of pneumonia during stay on intensive care was reached by the independent variables decompressive craniectomy (*p* = 0.0039 ; OR 4.45), hydrocephalus (*p* = 0.0028 ; OR 3.49), WFNS grade (*p* = 0.00017 ; OR 2.23) and age (*p* = 0.041 ; OR 1.03). Sex, shunting procedure, sepsis and diabetes insipidus did not show significant values (Table 4). For this analysis the Nagelkerke R Square value was 0.522 and the Hosmer and Lemeshow test value 0.618.

**Table 4:** Predictors of Pneumonia during stay on Intensive Care Unit

Risk factors	Odds ratio	95 % Confidence interval	p
Sex	0.78	0.36–1.68	0.52
Age	1.03	1.00–1.06	0.041
WFNS Grade	2.23	1.47–3.40	0.00017
Hydrocephalus	3.49	1.54–7.89	0.0028
Shunt	2.95	0.90–9.73	0.075
Sepsis	5.50	0.97–31.04	0.054
Diabetes insipidus	0.66	0.21–2.04	0.47
Decompressive craniectomy	4.45	1.61–12.26	0.0039

Binary logistic regression was performed as a multivariate analysis with dichotomized status of pneumonia (0 = “no pneumonia”; 1 = “pneumonia”) as the dependent variable and sex, age, WFNS grade, hydrocephalus, shunt, sepsis, diabetes insipidus and decompressive craniectomy as independent variables (covariates)

### Influence of complications and interventions on the occurrence of tracheostomy

With tracheostomy as the dependent variable and the complications and interventions on ICU as the independent variables binary logistic regression analysis was executed. Nagelkerke R Square (0.685) and the Hosmer and Lemeshow test (0.817) showed good values for our model regarding prediction of tracheostomy. Significant variables were WFNS grade (*p* = 0.043), pneumonia (*p* < 0.0001), sepsis (*p* < 0.031), shunting procedure (*p* = 0.00076) and decompressive craniectomy (*p* = 0.00041). Shunting procedure (OR 12.80), pneumonia (OR 10.02) and decompressive craniectomy (OR 8.28) had the strongest association regarding prediction of tracheostomy, followed by sepsis (OR 6.09). WFNS grade had a comparatively low impact on prediction of tracheostomy (OR 1.82). Sex, age, hydrocephalus and diabetes insipidus were no significant predictors of tracheostomy (Table 5).

**Table 5:** Factors leading to Tracheostomy during stay on Intensive Care Unit

Risk factors	Odds ratio	95 % Confidence interval	p
Sex	1.33	0.49–3.60	0.58
Age	0.99	0.96–1.03	0.74
WFNS Grade	1.82	1.02–3.23	0.043
Pneumonia	10.02	3.19–31.41	< 0.0001
Sepsis	6.09	1.18–31.55	0.031
Hydrocephalus	0.74	0.23–2.45	0.63
Shunt	12.80	2.90–56.47	0.00076
Diabetes insipidus	2.54	0.64–10.16	0.19
Decompressive craniectomy	8.28	2.57–26.71	0.00041

Binary logistic regression was performed as a multivariate analysis with dichotomized status of tracheotomia (0 = “no tracheostomy”; 1 = “tracheostomy”) as the dependent variable and sex, age, WFNS grade, pneumonia, hydrocephalus, shunt, sepsis, diabetes insipidus and decompressive craniectomy as independent variables (covariates)

## Discussion

With this study we analyzed the impact of complications and interventions in intensive care units on the outcome after one year in SAH. Further we found significant predictors of pneumonia and tracheostomy.

Decompressive craniectomy, sepsis and hydrocephalus were significant predictors for poor outcome and death after one year in our collective. Tracheostomies and shunting procedures were associated with survival after one year. Higher WFNS grade, hydrocephalus and decompressive craniectomy were predictors of pneumonia. Tracheostomy was associated with higher WFNS grade, pneumonia, sepsis, shunt procedure and decompressive craniectomy.

### Decompressive craniectomy and sepsis as predictors of poor outcome and death after one year

Decompressive craniectomy (outcome OR 21.77 ; death OR 6.17) and sepsis (outcome OR 14.67 ; death 6.08) were very strong predictors of poor outcome in our analysis.

Surgical decompressive craniectomy is a very invasive approach, but is also a potent manoeuvre to reduce intracranial pressure [24, 25]. It involves severe risks like hydrocephalus, external brain tamponade, sinking skin flap syndrome, seizures, cerebral haemorrhage and paradoxical brain herniation [26–30]. SAH patients suffering from the life-threatening effects of the SAH itself must then additionally overcome the debilitating decompressive craniectomy. Alexander et al. stated that decompressive hemicraniectomy in large middle cerebral artery stroke results in great reductions in mortality, but leads to survival of patients with severe or very severe disability. They judged the benefit of decompressive craniectomy to be questionable, if it reduces frequency of death but induces survival with severe permanent disability [28]. This is particularly a debatable issue in elderly patients [31, 32]. In analyzed SAH-sub collectives (43 of 744 patients and 79 of 939 patients) who underwent decompressive treatment, the rate of a favourable outcome of the decompressed patients was not convincing (25.6 % and 26.6 %), however the proportion of poor grade patients according to WFNS was very high (83.7 % and 77.2 %) [33, 34]. In our collective decompressed patients had a favourable outcome after one year in 11.9 % (5 of 42; one missing because no mRS data after one year was available) with exactly the same proportion of poor grade patients (83.7 %, 36 of 43) according to WFNS. One third of the decompressed patients died (15 of 42, 35.7 %, one missing). Accordingly, the majority of the decompressed patients survived with a severe to very severe disability (81.5 %, 22 of 27). Our results suggest that decompressive craniectomy strongly predicts death and poor outcome and support the concerns of Alexander et al. [28]. This emphasizes the debate, whether decompressive craniectomy is reasonable for these patients and raises the need for individual decisions by the treating specialists.

The time from the onset of elevated and uncontrollable intracranial pressure to the decompression treatment might be an important factor concerning the outcome [34]. The underlying reason (brain swelling, bleeding or infarction) for the decompressive craniectomy does not seem to be relevant for the rate of favourable outcomes [34].

Sepsis was described as a significant risk factor regarding the outcome after SAH in previous literature. Dasenbrock et al. report that central venous catheter associated infections were linked with increased likelihood of a poor outcome [16]. Similar statements made Frontera et al.: blood stream infection independently predicted death or severe disability at 3 months [17]. Taufique et al. summarized that poor physical quality of life was associated with sepsis and hydrocephalus after older age and pneumonia [9].

### Prediction of tracheostomy and prediction of survival through tracheostomy

Tracheostomy is a key intervention predicting the survival of the patient up to one year (OR 0.09 ; p = 0.0016), although it is not a significant predictor for the quality of life after one year (p = 0.11) in our cohort.

There is evidence, that tracheostomy has a direct beneficial impact on the survival of patients suffering from SAH. Ventilation time is subsequently reduced through tracheostomy. Ponfick et al. stated that every day of mechanical ventilation reduces the probability of a beneficial outcome and that tracheotomized patients with cerebrovascular diseases benefit from early in-patient rehabilitation, irrespective of the etiology of vascular brain injury [35]. The guidelines for the management of severe traumatic brain injury state that tracheostomy reduces mechanical ventilation days. However it does not alter rate of pneumonia or mortality in traumatic brain injury patients [36].

WFNS grade (OR 1.82 ; p = 0.043), pneumonia (OR 10.02 ; p < 0.0001), sepsis (OR 6.0 ; p = 0.031), shunting procedure (OR 12.80 ; p = 0.00076 ) and decompressive craniectomy (OR 8.28 ; p = 0.00041) were significant predictors of tracheostomy in our cohort. All of these variables come along with factors needing a longer ventilation time and therefore tracheostomy.

Szeder et al. analyzed patients with intracerebral hemorrhage and also found the initial clinical status (Glasgow Coma Scale) but also the radiological presence of hydrocephalus as predictors of need for tracheostomy [37]. Another study dealing with predictors of tracheostomy in patients suffering from intracerebral hemorrhage detected hydrocephalus to be a critical factor in prediction of tracheostomy, after volume of intracerebral hemorrhage, presence of chronic obstructive pulmonary disease (COPD) and ganglionic location of the hematoma [38]. In our collective only the subgroup of chronic hydrocephalus (shunting procedure), but not hydrocephalus was associated with tracheostomy.

### The role of hydrocephalus after SAH in the prediction of outcome and death after one year

Consecutive hydrocephalus after the subarachnoid hemorrhage determines the outcome and death after one year in a significant way (outcome: OR 3.71 ; *p* = 0.010 – death: OR 6.46 ; *p* = 0.00095). However, the subgroup of hydrocephalus-patients with chronic hydrocephalus, who required a shunting procedure had no significant effect on the outcome after one year (*p* = 0.96), but was more likely to survive the first year (OR 0.18, *p* = 0.025).

Without adequate treatment by implantation of an external ventricular drainage hydrocephalus is a life-threatening condition with increased intracranial pressure leading to an impaired clinical state. The consecutive decreased consciousness leads to complications like aspiration, pneumonia and thus extended mechanical ventilation. These factors associated with hydrocephalus might play a critical role in the determination of outcome in the early stage after SAH.

Our findings regarding the role of hydrocephalus on the outcome are well confirmed by the existing literature. Beneš et al. investigated the causes of poor outcome in patients with good-grade subarachnoid hemorrhage and found that the presence of hydrocephalus is a significant predictor of poor Glasgow Outcome Scale scores beneath the factor of age over 60 years [39]. Taufique et al. also saw a determination of outcome through the incidence of hydrocephalus in their cohort [9]. Galea et al. presented a large cohort of 3341 patients suffering from SAH, where the need for a cerebrospinal fluid diversion (OR 3.25 ; 95 % confidence interval (CI) 2.58–4.09 ; *p* < 0.001) was an independent predictor of an unfavourable outcome after increasing age, WFNS grade, preoperative rebleeding and delayed cerebral ischemia. They also discuss our mentioned idea of a direct effect of the hydrocephalus causing raised intracranial pressure, besides iatrogenic injury and re-hemorrhage [3].

On the one hand the subgroup of patients with chronic hydrocephalus, who required a shunting procedure more likely survived the first year (OR 0.18, *p* = 0.025) in our collective. Reasons might be beneficial pathomechanisms which come along with the implementation of a cerebrospinal fluid (CSF) drainage. They range from improvement of the clinical grade in poor grade SAH patients by treatment of the raised intracranial pressure to the theory, that CSF drainage can improve cerebral oxygenation and reduce delayed cerebral ischemia (DCI) [40, 41]. The question is whether these positive effects particularly take place in the subgroup of chronic hydrocephalus through a permanent CSF drainage and thereby lead to the survival of the patients.

On the other hand, chronic hydrocephalus and a consecutive shunting procedure had no significant positive influence on the quality of life after one year (*p* = 0.96) in our cohort. Regarding this point existing literature is quite heterogeneous. Wong et al. found that chronic hydrocephalus was significantly associated with poor quality of life after SAH [42]. Poon et al. examined the impact of chronic hydrocephalus on neurological outcome with a 6-month extended Glasgow Outcome Scale score in a more differentiated manner. They detected an association of chronic hydrocephalus with a better extended Glasgow Outcome Scale score among patients with a poor WFNS grading, but in patients with a good WFNS grading, chronic hydrocephalus was associated with a poor extended Glasgow Outcome Scale score [43].

### The role of pneumonia and diabetes insipidus in the prediction of outcome and death after one year

In our cohort pneumonia (p = 0.069 respectively p = 0.46) and diabetes insipidus (p = 0.19 respectively p = 0.11) were no significant predictors of outcome or death after one year. This is in line with a publication of Wartenberg et al. who analyzed 580 patients and had similar outcome data to those of our cohort. Fever, anemia and hyperglycemia were associated with mortality and poor functional outcome, but not pneumonia and diabetes insipidus [18]. However, there are some publications which found a significant association between pneumonia and reduced outcome or poor quality of life [9, 16, 17]. To our knowledge there is no publication describing a significant association between poor outcome after SAH and diabetes insipidus.

### WFNS grade and age as predictors of poor outcome and death after one year

WFNS grade (outcome OR 3.86 ; death OR 4.67) and age (outcome OR 1.06 ; death OR 1.10) are known predictors of outcome, which have been confirmed in several studies before [4, 7–9].

### Standardization of management and outcome measurement after aneurysmal SAH

In order to minimize varieties of management of cases of aneurysmal SAH, standardized management protocols (SMPs) are useful as they are assumed to improve patient outcomes after subarachnoid hemorrhage. Therefore, outcome measurement, management of acute SAH, early brain injury, DCI and general neurocritical care should be standardized.

A systematic review of Taran et al. aimed to detect the impact of SMPs after SAH [44]. Two randomized control trials and 35 observational studies were therefore included. It was remarkable, that significant limitations like single-center case series with small patient sizes and inconsistent definitions of key terms and outcome reporting practices inhibited a meta-analysis for 6-month mortality and neurologic outcomes and therefore the anticipated effect of SMPs could not be confirmed [44].

In order to overcome systematic issues of incomparability regarding outcome measurement, common data elements have been defined regarding outcomes and endpoints [19]. In these terms the modified Rankin Scale score is recommended, while Death, Glasgow Outcome Scale score, and Glasgow Outcome Scale-extended were classified as supplemental. Timing for long-term outcome assessment is advised to be conducted at 12 months [19].

There are lacking standards regarding ventilation-, monitoring- and sedation-management after aneurysmal SAH, which are also assumed to have effects on the outcome [45]. With the help of a questionnaire ICU practices in aneurysmal SAH in Germany were evaluated and compared to guidelines, if existing. In this study, ICU management after aneurysmal SAH was found to be very heterogeneous. This leads to the conclusion, that evidence in this area of SAH-management is insufficient [45]. There is a necessity to define optimal requirements and guidelines of ICU management after SAH, particularly management of ventilation, timing of tracheostomy, sedation and intracranial pressure (ICP) / cerebral metabolism monitoring.

## Limitations

The higher probability of survival of tracheotomized and shunted patients after one year might be a simple result of the imperative of survival regarding the first critical days after SAH. Only patients who survive will get a tracheostomy or shunting procedure. Parts of the data regarding complications and interventions were collected retrospectively. Moreover, our results might have a limited range concerning a generalization, because data was collected at a single-center in middle Europe. Specialization regarding the treatment of SAH and its complications, as well as differences concerning the choice of treatment modalities, treatment capacities and logistical limitations in comparison to other hospitals might therefore lead to a bias regarding the generalizability of these observations. More powerful prospective multicenter randomized trials with high patient numbers, which adhere to standardization of outcome measurement and management of aneurysmal SAH, will be needed to confirm our results.

## Conclusions

Beside baseline characteristics (WFNS-grade and age) decompressive craniectomy, sepsis and hydrocephalus strongly influence the outcome and survival in SAH after one year. Tracheostomy and chronic hydrocephalus in terms of a consecutive shunting procedure did not significantly influence the quality of life after one year, but these patients were more likely to survive the first year after SAH. Several factors with influence on the clinical outcome of patients cannot be controlled by therapy. But rapid and decisive treatment of infections, intracranial perfusion and intracranial pressure might influence the frequency of critical complications and interventions and accordingly might lead to a more favorable outcome after SAH. The significance of decompressive craniectomy in SAH seems to be debatable in terms of outcome after one year.

## Data Availability

The datasets generated and/or analysed during the current study are not publicly available due to further analysis and publication but are available from the corresponding author on reasonable request.

## Abbreviations

95% CI: 95% confidence interval
COPD: Chronic obstructive pulmonary disease
CSF: Cerebrospinal fluid
CT: Computed tomography
DCI: Delayed cerebral ischemia
EVD: External ventricular drainage
ICP: Intracranial pressure
ICU: Intensive care unit
mRS: Modified rankin scale
OR: Odds ratio
SAH: Subarachnoid hemorrhage
SMPs: Standardized management protocols
WFNS: World Federation of Neurosurgical Societies
